# Effect of Surface Roughness Characteristics on Structural Performance of Hollow Core Slabs

**DOI:** 10.3390/ma14102610

**Published:** 2021-05-17

**Authors:** Yong-Jun Lee, Hyeong-Gook Kim, Chan-Yu Jeong, Dong-Hwan Kim, Sang-Pil Han, Kil-Hee Kim

**Affiliations:** 1 Department of Architectural Engineering, Kongju National University, 1223-24 Cheonandaero, Seobuk, Cheonan 31080, Korea; lyj8315@kongju.ac.kr (Y.-J.L.); anthk1333@kongju.ac.kr (H.-G.K.); ssbachany@kongju.ac.kr (C.-Y.J.); kimdh@kongju.ac.kr (D.-H.K.); 2 Department of Fire Protection Engineering, Sangji University, 83, Sangjidaegil, Wonju 26339, Korea; hsfeel@sangji.ac.kr; 3 Department of Architectural Engineering & Urban Systems Engineering, Kongju National University, 1223-24 Cheonandaero, Seobuk, Cheonan 31080, Korea

**Keywords:** flexural strength, hollow core slabs, horizontal shear strength, prestressed concrete, surface roughness

## Abstract

This study was conducted to evaluate the flexural performance of hollow core slabs (HCS) incorporating the effect of surface roughness. The HCSs are suitable for long span structures due to reduced self-weight. The specimens were HCS with topping concrete and the variables were cross sectional height and surface roughness. The tests were conducted on simply supported beams under four-point loads. The results showed that specimens with interface roughness applied in the lengthwise direction of members exhibited ductile flexural behavior up to peak load than those with interface roughness applied in the member width direction. Their flexural strength was also higher by 1–7% on average, indicating that they are advantageous in improving structural performance.

## 1. Introduction

Society is changing rapidly with the development of technology and information, and the construction industry is exerting efforts to deviate from traditional labor-intensive methods. Against this backdrop, methods and structural systems that make on-site management convenient and minimize the number of on-site tasks have come under the spotlight. Structural members are gradually becoming more simplified and they are expected to be applied to structures in the form of affordable modules.

Prestressed concrete hollow core slabs (HCS) are members with reduced self-weight due to the hollow core formed in the center of the cross section, providing enhanced structural efficiency. The HCSs are widely used across Europe and North America because their upper and lower tendons allow them to have flexural performance superior to that of reinforced concrete slabs [1,2,3,4,5,6,7,8]. Compared to general cast-in-place reinforced concrete structures, HCSs can shorten construction periods and facilitate on-site management because they are assembled on-site after being manufactured in factories. The reduced self-weight due to the hollow core makes them more advantageous for long span structures and they are thus actively used in long span floor structures of facilities, such as parking lots, distribution centers, discount stores, and semiconductor factories [9,10]. The HCS has shown outstanding performance in absorbing heavyweight impact noise, a major factor of interlayer noise and will contribute to lessening disputes over interlayer noise by reducing vibration when applied to multi-unit dwellings [11].

However, several factors must be considered when applying the HCS on-site. Since the HCS members are manufactured through extrusion of high strength concrete, which has low slump on a long line bed of 90–180 m, shear reinforcement arrangement is difficult and shear capacity must be assessed in consideration of the thin webs arising from the hollow cores [2,4,11,12,13,14]. In addition, consideration of the floor deformation performance, according to lateral forces such as earthquakes is also required [15,16]. When applied to structures, there should be continuity between the slab ends so that the entire floor engages in diaphragm action. For the hollow core slabs with topping concrete, interfaces must be structurally assessed to prevent sliding failure in the horizontal direction, so as to allow composite cross sections to exhibit the expected structural performance [3,8,17,18,19,20,21]. In push-off tests with manufacturing method and interface roughness as variables, Mones and Breña (2013) [3] found that horizontal shear strength evaluation was significantly influenced by the presence of laitance, the interface roughness, and the direction. Tassios and Vintzeleou (1987) [19] and Gohnert (2000) [20], based on experiments, proposed equations to predict horizontal shear strength by analyzing the load transfer mechanisms in relation to differences in interface roughness. Beushausen (2001) [21] considered reinforcement amount as a key factor in evaluation of horizontal shear strength and demonstrated through experiments that satisfactory performance was achieved from interface roughness alone. In addition, Santos and Júlio (2012) [22] compared the studies of previous researchers on shear friction and analyzed the main influencing factors at the interface and reported the necessity of considering the shrinkage and stiffness of the concrete-to-concrete interface.

In previous studies [3,8,17,18,19,20,21,22], most researchers have examined the shear capacity of the HCS with topping concrete in terms of interface roughness. The HCSs are manufactured such that the roughness applied to the upper surface is in the direction of product width (horizontal) in order to secure shear strength. However, for interfaces to secure horizontal shear strength, one must examine the effect of surface roughness not only on shear capacity, but also on flexural behavior, such as increase flexural strength and ductility.

As such, to evaluate the effect of the interface roughness of HCS with topping concrete on structural performance, this study conducted flexural tests with cross sectional height and interface roughness direction as variables. The experimental results were compared to predictions based on design criteria [23,24,25,26] to verify the horizontal shear strength needed to meet quantitative requirements of flexural strength and structural performance.

## 2. Major Structural Design Criteria of the HCS

### 2.1. Flexural Strength

Various design codes [23,24,25,26] present the calculation of flexural strength of prestressed concrete members based on ultimate strength design. The cracking moment (*M_cr_*) of prestressed concrete members that allow cracks to form, including HCS, can be obtained as:

(1) $$M_{cr}=f_{r}S_{b}+P_{e}\left(\frac{S_{b}}{A_{c}}+e\right)$$

here, $f_{r}$ is the flexural crack strength of concrete, $S_{b}$ is the section modulus from the neutral axis to the lower surface of the cross-section, $P_{e}$ is the effective jacking force of tendons, $A_{c}$ is the cross-sectional area of concrete, and e is the distance from the neutral axis of the cross-section to the tendon center.

The nominal flexural strength (*M_n_*) of the HCS depends on calculation of the tendon stress (*f_ps_*). Strain compatibility conditions are used when $f_{ps}<0.5f_{pu}$ and tendon stress conditions when $f_{ps}\geq 0.5f_{pu}$. Considering the use of bonded tendons and a value of $f_{ps}\geq 0.5f_{pu}$ of general HCS, the stress of bonded tendons and nominal flexural strength can be calculated by Equations (2) and (3), respectively:

(2) $$f_{ps}=f_{pu}\left[1-\frac{\gamma_{p}}{\beta_{1}}\left\{\rho_{p}\frac{f_{pu}}{f_{c}^{\prime }}+\frac{d_{s}}{d_{p}}\left(\omega -\omega^{\prime }\right)\right\}\right]$$

(3) $$M_{n}=\left[A_{ps}f_{ps}\left(d_{p}-\frac{a}{2}\right)+A_{s}f_{y}\left(d_{s}-\frac{a}{2}\right)\right]$$

here, $f_{pu}$ is the tensile strength of the tendons, $\beta_{1}$ is the equivalent stress block coefficient, $\rho_{p}$ is the tendon ratio ($\rho_{p}=A_{ps}/bd_{p}$), ω is the steel index of tension reinforcement ($\omega =\rho f_{y}/f_{c}^{\prime }$), $\omega^{\prime }$ is the steel index of compressive reinforcement ($\omega^{\prime }=\rho^{\prime }f_{y}/f_{c}^{\prime }$), ρ is the ratio of tension reinforcement ($\rho =A_{s}/bd_{s}$), $\rho^{\prime }$ is the ratio of compressive reinforcement ($\rho^{\prime }=A_{s}^{\prime }/bd_{s}$), $A_{ps}$ is the cross-sectional area of the tendons, $A_{s}$ is the cross-sectional area of tension reinforcement, $A_{s}^{\prime }$ is the cross-sectional area of compressive reinforcement, $f_{y}$ is the yield strength of reinforcement, $f_{c}^{\prime }$ is the compressive strength of concrete, b is the member width, $d_{p}$ is the distance from the compressive edge to the center of the tendon cross-section, $d_{s}$ is the distance from the compressive edge to the center of the cross- section of tension reinforcement, a is the depth of the equivalent stress block, and $\gamma_{p}$ is the coefficient determined by tendon type. In KCI 2017 [23], ACI 318-19 [24], and the PCI Design Handbook [25], $\gamma_{p}=0.55$ is proposed for $f_{py}/f_{pu}\geq 0.80$, $\gamma_{p}=0.40$ for $f_{py}/f_{pu}\geq 0.85,$ and $\gamma_{p}=0.28$ for $f_{py}/f_{pu}\geq 0.90$. In Equation (2), $\left\{\rho_{p}\frac{f_{pu}}{f_{c}^{\prime }}+\frac{d_{s}}{d_{p}}\left(\omega -\omega^{\prime }\right)\right\}$ must be greater than 0.17, considering the compressive reinforcement, and the distance from the compressive edge to the center of the cross- section of the compressive reinforcement should be set below $0.15d_{p}$. The design flexural moment ($M_{u}\left(=\phi M_{n}\right)$) must be at least 1.2 times the crack moment ($M_{cr}$), according to KCI 2017, ACI 318-19, and the PCI Design Handbook, and at least 1.15 times the crack moment ($M_{cr}$) according to EC 2 [26] to induce ductile failure, in which cracks are allowed.

### 2.2. Horizontal Shear Strength

The design codes and guidelines of various countries [23,24,25,26] specify that the design horizontal shear strength of prestressed concrete members must be greater than the factored shear strength, as shown in:

(4) $$V_{u}\leq \phi V_{nh}$$

here, $V_{u}$ is the factored shear strength, $V_{nh}$ is the design horizontal shear strength, and ϕ is the capacity reduction factor.

KCI 2017, ACI 318-19, and the PCI Design Handbook classify the design horizontal shear strength into three types, depending on surface roughness and presence of a minimum shear connector. The first type (1) has less than the minimum required shear connection and is characterized by a clean contact surface with no suspended particles and an intentionally roughened surface. The second (2) has greater than the minimum required shear connection, a clean contact surface with no suspended particles and a surface that was not intentionally roughened. The third (3) has greater than the minimum required shear connection, a clean contact surface with no suspended particles and a surface that was intentionally roughened and has a depth of approximately 6 mm. The horizontal shear strength of the first and second types is calculated by Equation (5) and that of the third type by Equation (6):

(5) $$V_{nh}\leq 0.56b_{v}d_{p}\;(\mathrm{ACI}\;318-19\;\mathrm{and}\;\mathrm{the}\;\mathrm{PCI}\;\mathrm{Design}\;\mathrm{Handbook},\;V_{nh}\leq 0.55b_{v}d_{p})$$

(6) $$V_{nh}=\left(1.8+0.6\rho_{v}f_{yt}\right)\lambda b_{v}d_{p}$$

here, $b_{v}$ is the width of the interface cross-section, $\rho_{v}$ is the reinforcement ratio of shear connector, $f_{yt}$ is the yield strength of shear connector, and λ is the coefficient of lightweight concrete. The values must be calculated according to the shear friction design method if the factored shear strength ($V_{u}$) obtained by Equation (6) exceeds $\phi 3,5b_{v}d_{p}$.

**Figure 1:**
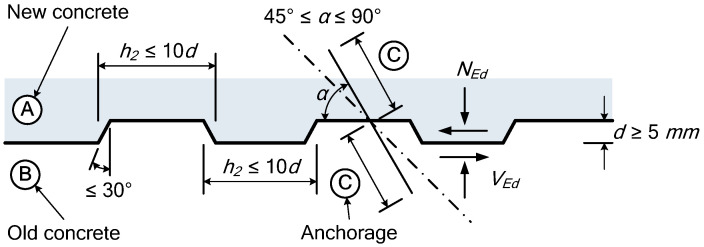
Indented construction joint [26].

As shown in Equation (7), The components for the EC 2 [26] approach are the cohesion component ($cf_{ct}$), the friction component ($\mu \sigma_{n}$), and the component due to the shear connector. When considering only the cohesion component ($cf_{ct}$) in Equation (7) and excluding the contributions of the shear connector and compressive stress, the four surface types are as shown in Table 1. Compared to KCI 2017, ACI 318-19 and the PCI Design Handbook, EC 2 presents more specific details of interfacial horizontal shear strength and the indented construction joint, as shown in Figure 1.

(7) $$V_{nh}=cf_{ct}+\mu \sigma_{n}+\rho_{v}f_{yt}\left(\mu sin\alpha +cos\alpha \right)<0.5\nu f_{cd}$$

here, c and μ are the coefficients expressing the interfacial state, $f_{ct}$ is the design tensile strength of concrete, $\sigma_{n}$ is the compressive stress acting vertically across the entire cross section, α is the shear connector angle, ν is the strength softening factor, and $f_{cd}$ is the design compressive strength of concrete.

## 3. Experimental Program

### 3.1. Materials

To evaluate the structural performance of the HCS in consideration of topping concrete, two types of concrete used to fabricate topping concrete and HCS specimens were mixed with target design strengths of 27 and 49 MPa, respectively. The coarse aggregates used in the concrete mix were crushed aggregates with a maximum size of 19 mm and crushed sand with a maximum size of 5 mm for fine aggregates. The coarse and fine aggregates had specific gravity values under oven-dry of 2.57 and 2.59 g/cm^3^, and absorption rates of 1.07% and 1.10%, respectively.

**Figure 2:**
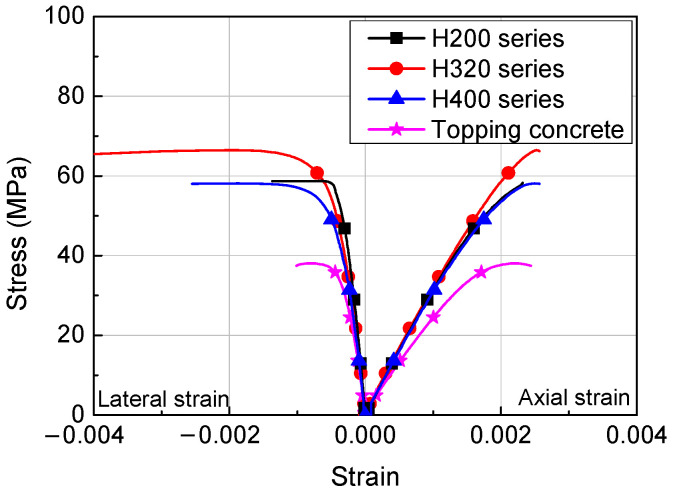
Stress-strain relationships of concrete.

Eighteen concrete cylinders, measuring Φ100 mm × 200 mm, were fabricated for testing to estimate the compressive strength of HCS and topping concrete and the curing was performed under the same conditions as specimens [27,28]. The compressive strength of concrete was matched to failure of each specimen and the results are shown in Table 2 and Figure 2. During the tests, the age of concrete is 28–32 days for the HCS and 21–25 days for topping concrete, according to the experimental schedule. The stress-strain relationship of concrete shown in Figure 2 was obtained from readings of strain gauges attached in the longitudinal and lateral directions of the concrete cylinders. The compressive strength of concrete is shown in Table 2. For HCS, the average compressive strengths were 56.6 MPa for the H200 series, 51.8–62.6 MPa for the H320 series, and 51.7 MPa for the H400 series. All average compressive strength values were higher than the design strength. The average compressive strengths of topping concrete were 38.9 MPa for the H200 series, 32.1–33.7 MPa for the H320 series, and 35.9 MPa for the H400 series.

To secure the cross-sectional performance expected of the HCS, this study used prestressed strands with diameters (Φ) of 12.7 and 9.5 mm to fabricate the specimens [29,30]. The yield strength and tensile strength of the strands, as provided by the manufacturer, were 1581 and 1860 MPa, respectively.

### 3.2. Specimens

**Figure 3:**
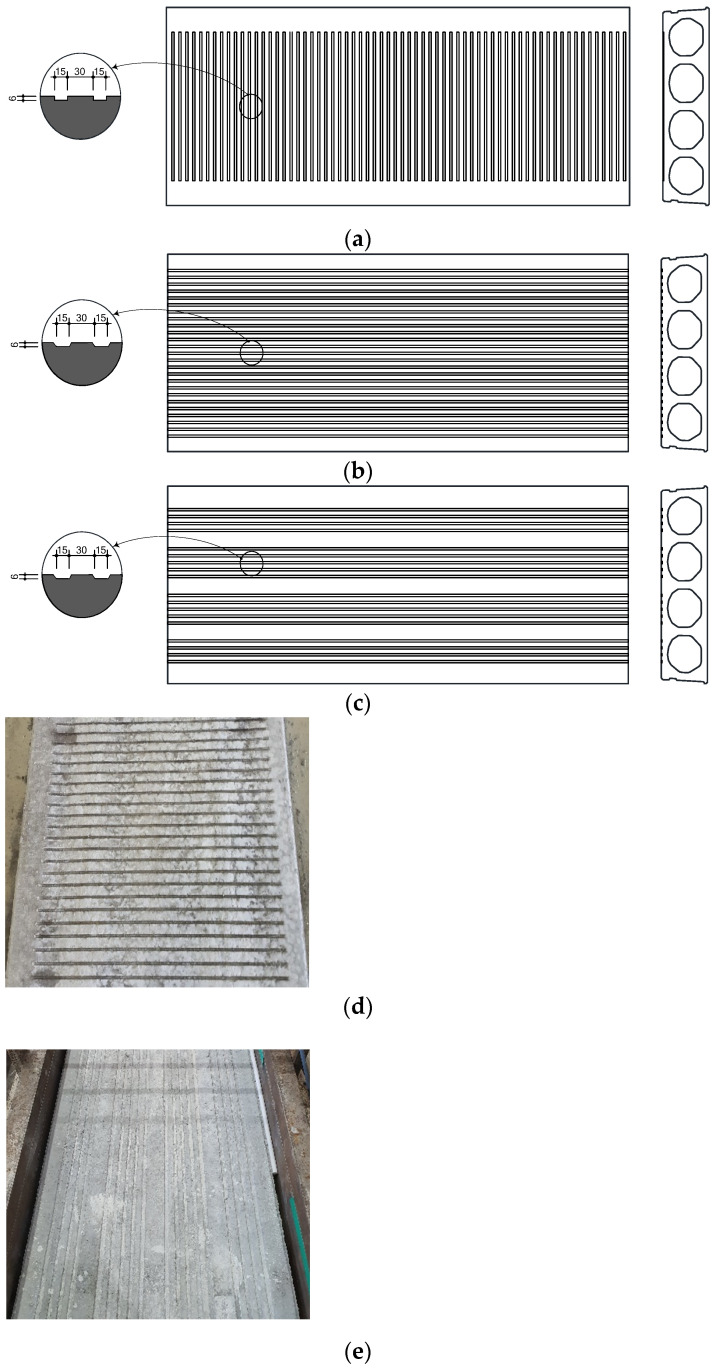
Upper surface of specimens (unit: mm): (**a**) CF series; (**b**) CC series; (**c**) CN series; (**d**) photograph of CF series; (**e**) photograph of CN series.

To evaluate the structural performance of the HCS in consideration of topping concrete, the specimens were designed with height (*h*) and interface roughness as variables, as shown in Table 2. In the specimen names given in Table 2, as shown in Figure 3, CF represents specimens with grooves applied in the width (horizontal) direction at a depth of 6 mm on the interface; CC represents grooves applied in the lengthwise direction at a width of 30 mm and depth of 6 mm on the interface; CN refers to specimens having the same interface roughness as CC, but fewer in number by 28%. The height of HCS is represented by numbers 200, 320, and 400.

**Figure 4:**
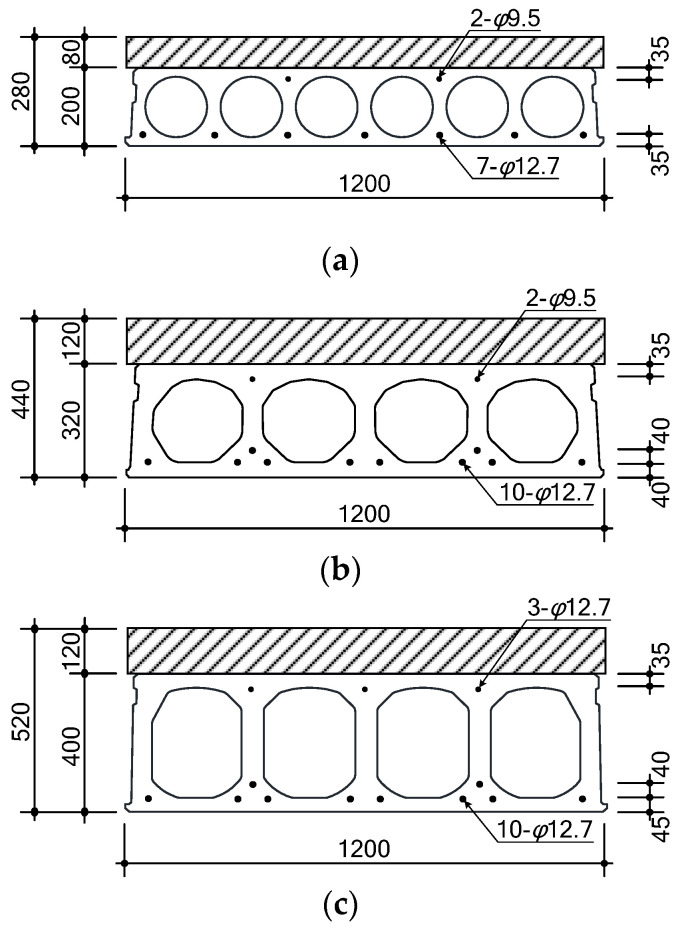
Details of cross-section (unit: mm): (**a**) CF200 and CN200; (**b**) CF320 and CC320 and CN320; (**c**) CF400 and CN400.

**Figure 5:**
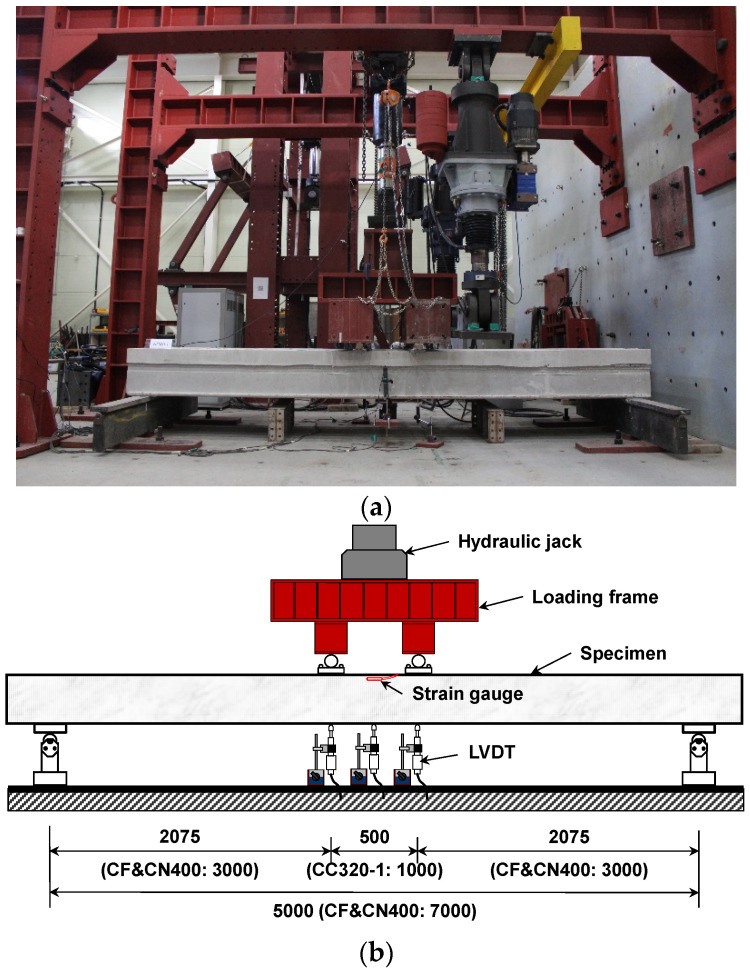
Test setup of specimen (unit: mm): (**a**) photograph; (**b**) elevation view.

As shown in Table 2, Figure 4 and Figure 5, the specimens had cross-sections of 1200 mm × 280 (440, 520) mm, lengths of 5000 (7000) mm and pure spans of 4650 (6500) mm. The height of the topping concrete in relation to the HCS cross-section was designed to be 80 mm for CF200 (CN200), 120 mm for CF320 (CC320, CN320), and 120 mm for CF400 (CN400). All specimens exhibited dominant flexural behavior with a shear span to depth ratio (*a/d*) in the range of 4.65–8.47, which prevented shear failure from occurring before flexural behavior.

To reflect the preferred on-site arrangement in relation to the HCS cross-section, CF200 (CN200) had strands as tendons [29,30], with 7-Φ12.7 arranged at the bottom and 2-Φ9.5 at the top. CF320 (CC320, CN320) had 10-Φ12.7 at the bottom of the cross-section and 2-Φ9.5 at the top. Using Φ12.7, CF400 (CN400) had 10-Φ12.7 at the bottom of the cross-section and 3-Φ12.7 at the top.

Shear reinforcement was not considered for any of the specimens, so as to all the conducting of flexural tests to examine the effect of specimen height and interface roughness on structural performance. When fabricating the specimens, jacking force was set at 65% of the tendon tensile strength. While reinforcement was arranged and integrated before casting topping concrete to ensure continuity of HCS slabs during the on-site application, this study considered only topping concrete in order to evaluate structural performance in relation to interface roughness.

### 3.3. Loading and Measurement Methods

As shown in Figure 5, the flexural tests were performed on simply supported beams under the four-point loads using a 1000 kN hydraulic jack for loading. The load control method was employed and loads were applied at a rate of 0.5 kN/sec. The experiment was terminated when the load dropped below 95% after the peak load in order to perform a stable experiment due to the influence of the prestressing. As shown in Figure 5, an LVDT (linear variable differential transformer) was installed at the central bottom, where the most deformation occurs, to measure the deflection of specimens under loading. At the compressive edge between the loading points, strain gauges for concrete were attached to measure concrete strain.

## 4. Experimental Results and Analysis

### 4.1. Load-Deflection Relationship

**Figure 6:**
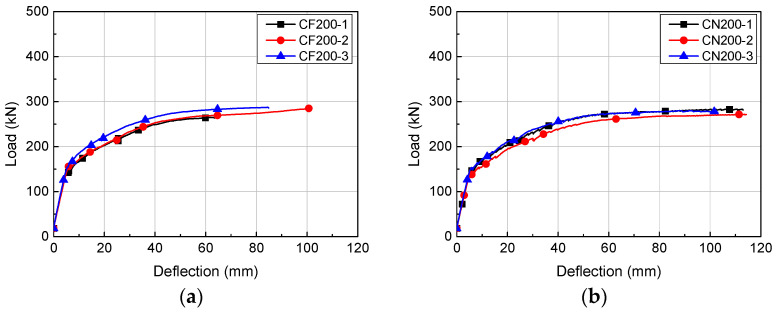
Load-deflection relationships of H200 series: (**a**) CF200; (**b**) CN200.

**Figure 7:**
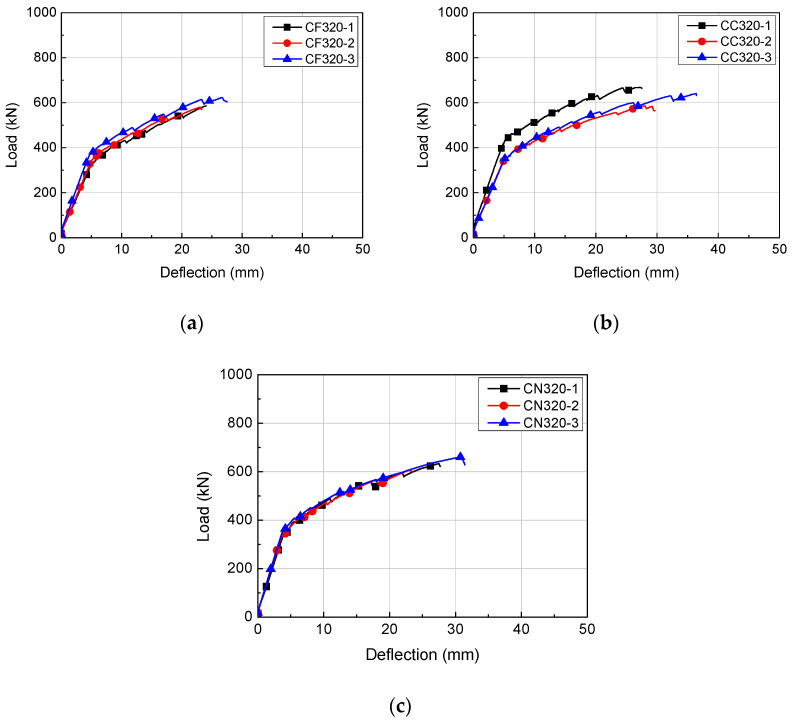
Load-deflection relationships of H320 series: (**a**) CF320; (**b**) CC320; (**c**) CN320.

**Figure 8:**
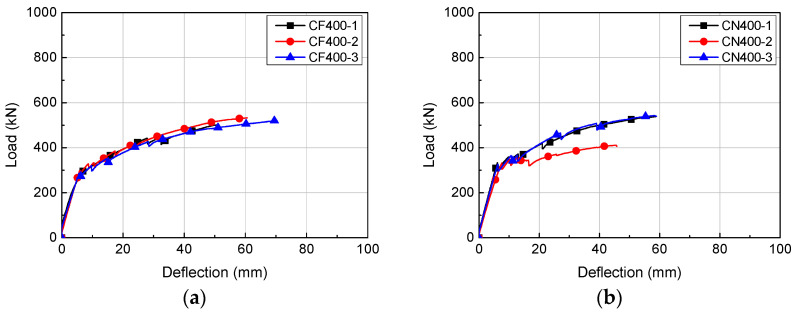
Load-deflection relationships of H400 series: (**a**) CF400; (**b**) CN400.

The load-deflection relationships of the HCS with topping concrete are presented in Figure 6, Figure 7 and Figure 8. The load of each specimen was obtained from a load cell attached on the hydraulic jack and deflection was the displacement measured from the LVDT at the bottom of the specimens. Regardless of the cross-sectional height and interface roughness, HCS with topping concrete exhibited linear behavior until the first flexural crack formed. Linear behavior of a certain gradient continued after the formation of flexural cracks with increasing load, but flexural stiffness was lower than at initial loading, when flexural cracks were not yet formed. The stiffness after the formation of flexural cracks was higher in specimens with smaller shear span to depth ratios (*a/d*).

The experimental results of the HCS with topping concrete are shown in Table 3. For CF200, flexural cracks formed at an average of 152.9 kN and deflection averaged to 5.52 mm. The peak load of CF200 was 278.9 kN on average and the corresponding deflection was 81.90 mm. The load and deflection were 147.0 kN and 5.70 mm on average at the point of flexural crack formation and 283.9 kN and 103.71 mm on average at peak load.

For CF320, flexural cracks formed at an average load of 354.8 kN and deflection averaged 5.16 mm. The peak load of CF320 was 595.8 kN on average and the corresponding deflection was 24.56 mm. During the formation of flexural cracks, CC320 and CN320 were under average loads of 382.5 kN and 363.2 kN, and deflections were 5.24 mm and 4.13 mm, respectively. The average peak loads of CC320 and CN320 were 631.2 kN and 633.5 kN, and the deflections were 30.55 mm and 27.20 mm, respectively. Compared to other specimens, as shown in Figure 7b, CC320-1 had the highest flexural rigidity because it was designed to have a shear span to depth ratio (*a*/*d*) of 4.65, while the other specimens had a higher ratio of 5.19.

During the formation of flexural cracks, the load and deflection of CF400 averaged 295.5 kN and 6.28 mm, respectively. The load and deflection at peak load were 518.0 kN and 60.49 mm on average. The load and deflection of CN400 during the flexural crack formation were 329.3 kN and 6.20 mm on average, while the same values at peak load averaged 539.9 kN and 58.13 mm, respectively.

**Figure 9:**
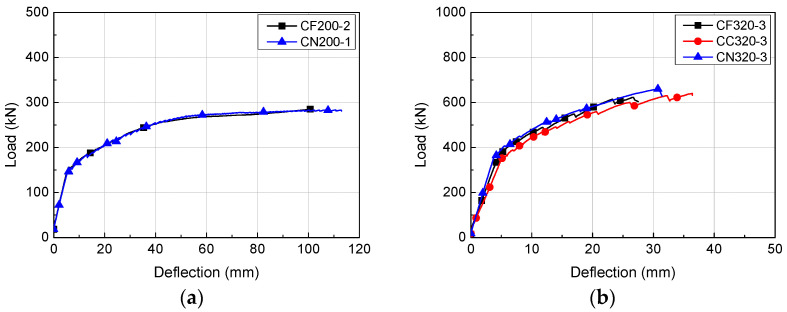

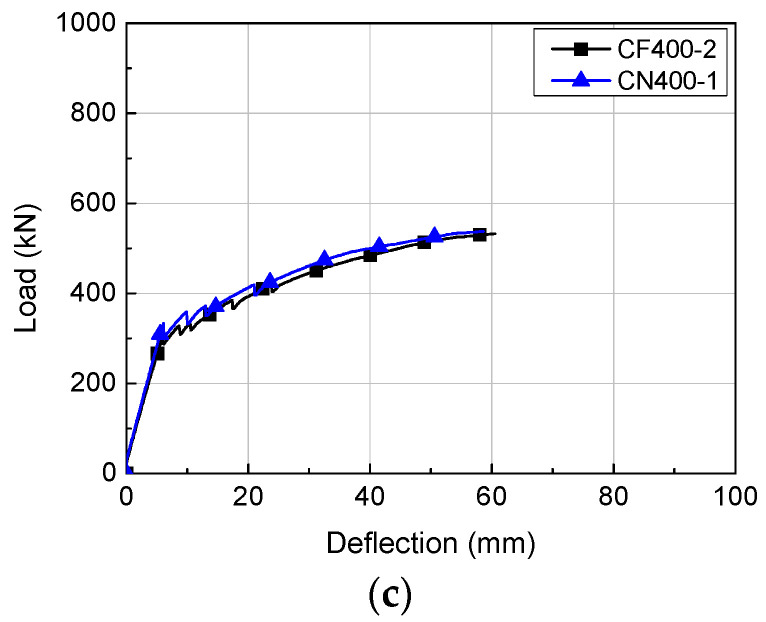
Comparison of load-deflection relationships according to the surface roughness directions: (**a**) H200 series; (**b**) H320 series; (**c**) H400 series.

As shown in Figure 9 and Table 3, HCS with topping concrete exhibited similar behavior regardless of cross-sectional height and interface roughness. However, a comparison of specimens using deflection at peak load showed that CN200 had higher peak deflection by 27% on average compared to CF200 of the same series. Even if not considering the error of shear span to depth ratio of CC320-1, the peak deflection was higher for CC320 by about 24% compared to CF320, while that of CN320 was higher by about 11% compared to CN320. CC320 had superior peak deflection by about 12% on average over CN320, which was the specimen with number of grooves reduced by 28%. This showed that peak deflection can be enhanced by applying grooves in the length direction of members or by increasing the number of grooves. CN400 showed flexural behavior similar to that of CF400, indicating that structural performance requirements can be satisfied by reducing the number of grooves by 28% if interface roughness is applied in the length direction. The deformation at peak load was about 4% lower for CN400 than for CF400.

### 4.2. Cracking Patterns

**Figure 10:**
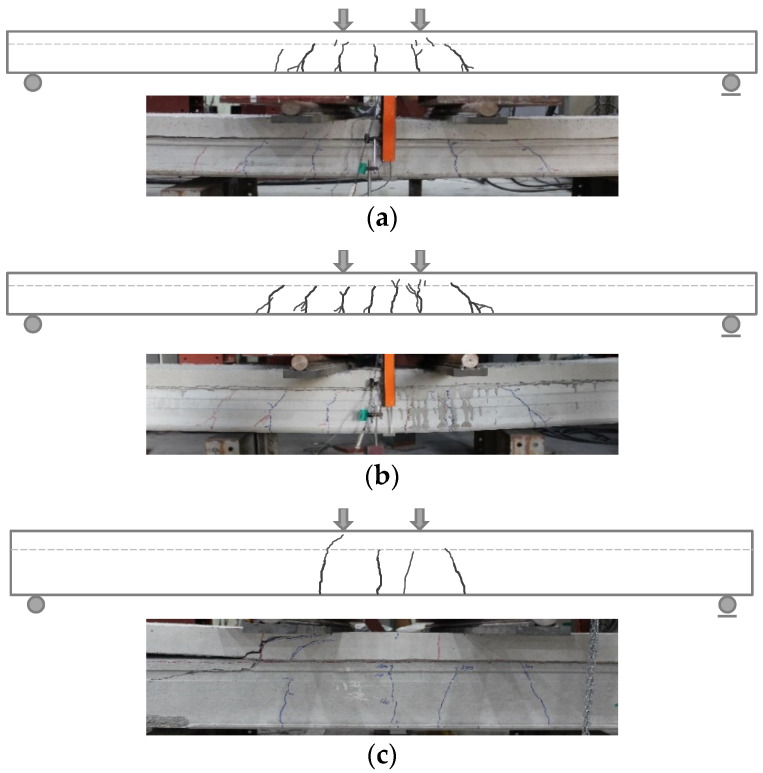

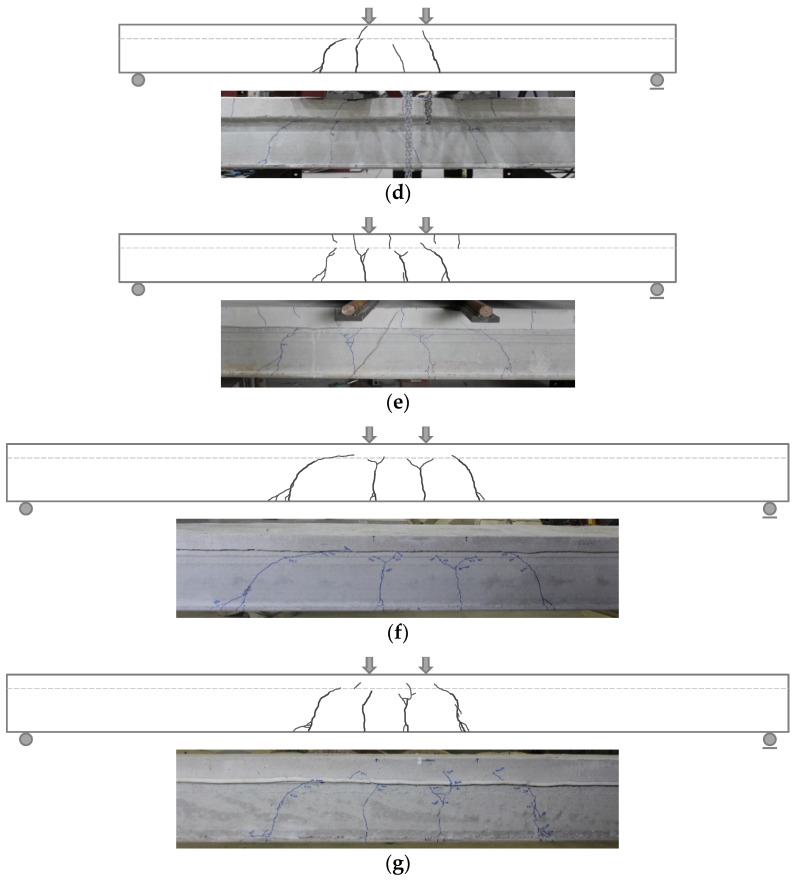
Crack patterns of specimens at peak load: (**a**) CF200-1; (**b**) CN200-1; (**c**) CF320-3; (**d**) CC320-3; (**e**) CN320-3; (**f**) CF400-1; (**g**) CN400-1. The photographs were the crack patterns of specimens at the end of the loading.

Figure 10 shows the crack patterns of the HCS specimens with topping concrete at peak load. The crack patterns of major specimens shown in Figure 10 were in the form of flexural cracks in the tensile area between the loading points at initial loading and moved towards the compressive edge and slab ends with increasing load. As shown in Table 3, due to the prestressed strands and composite cross sections, flexural cracks occurred at average loads of 147.0−152.9 kN for the H200 series, 354.8−382.5 kN for the H300 series and a high 295.5−329.9 kN for the H400 series. After the peak load, some cracks widened significantly and caused a drop in stress. As shown in Figure 10, similar crack patterns were observed regardless of direction of interface roughness. Among the H320 series, which was designed to have the smallest shear span to depth ratio (*a*/*d*), horizontal shear cracks were observed in some CF320 specimens with interface roughness applied in the member width direction, but not in CC320 and CN320 specimens with interface roughness applied in the member length direction.

### 4.3. Flexural Strength

Table 3 provides a comparison of experimental results of flexural tests on the HCS with topping concrete and predicted flexural strength based on design codes. The experimental moment value was calculated using load measurements from the load cell attached on the hydraulic jack. The predicted results were calculations of flexural strength based on KCI 2017 [23] and ACI 318-19 [24]. For more precise analysis, the actual measurements of topping concrete were obtained and reflected in the results as shown in Table 3.

As shown in Table 3, the experimental crack moment (*M_cr_*) of the H200 series averaged 158.6 kN·m for CF200 and 152.5 kN·m for CN200. The predicted average crack moment was 164.0 kN·m for CF200 and 165.7 kN·m for CN200, which translates to experimental-to-predicted ratios of 0.97 and 0.92, respectively. For the H320 series, the average crack moment values obtained experimentally were 368.1 kN·m and 378.5 kN·m for CF320 and CC320, respectively. The predicted crack moment values were 406.4 kN·m and 390.6 kN·m, translating to experimental-to-predicted ratios of 0.91 and 0.97, respectively. The average experimental and predicted crack moments of CN320 were 376.8 kN·m and 396.8 kN·m, which gives a ratio of 0.95. For the H400 series, the average crack moments based on experimental results were 443.2 kN·m for CF400 and 494.0 kN·m for CN400, and the predicted crack moments were 436.2 kN·m and 446.8 kN·m, respectively. That is, the experimental-to-predicted ratios were 1.02 and 1.11, respectively. The reason why the crack moment ratio of the H200 and H320 series is lower than the H400 series that some cracks bear the initial load.

As shown in Table 3, the nominal flexural strength (*M_n_*) of the H200 series obtained from experiments was on average 289.3 kN·m for CF200 and 294.5 kN·m for CN200. The predicted nominal flexural strength averaged 272.3 kN·m and 275.1 kN·m, giving ratios of 1.06 and 1.07, respectively. For the H320 series, the experimental nominal flexural strengths were 618.2 kN·m for CF320 and 627.0 kN·m for CC320. The predicted nominal flexural strengths were 632.8 kN·m and 612.1 kN·m, which translate to experimental-to-predicted ratios of 0.98 and 1.03, respectively. The average experimental and predicted nominal flexural strength values of CN320 were 657.3 kN·m and 628.5 kN·m, translating to a ratio of 1.05 on average. For the H400 series, the average nominal flexural strengths based on experiments were 777.1 kN·m for CF400 and 809.9 kN·m for CN400. The predicted nominal flexural strengths were 732.1 kN·m and 749.0 kN·m, which give ratios of 1.06 and 1.08, respectively.

As shown in Table 3, experimental values of flexural strength were compared to predictions based on KCI 2017 and ACI 318-19. The average experimental-to-predicted ratio was in a range of 0.98−1.08, indicating that flexural strength requirements were met. The predictions were found to be fairly accurate for HCS with topping concrete, regardless of the cross-sectional height and interface roughness. The CN series, which achieved interface roughness using grooves in the member length direction, had flexural strength higher by 1–7% compared to the CF series, which achieved interface roughness using grooves in the member width direction. The CN series showed satisfactory structural performance even when the number of grooves was reduced by 28%. In addition, flexural strength values obtained from experiments were higher than crack moment values by 1.64–1.93 on average. This satisfied the requirement of flexural moment having to be at least 1.2 times larger than crack moment in order to induce ductile failure, as specified in KCI 2017 and ACI 318-19.

### 4.4. Horizontal Shear Strength Review

To review the horizontal shear strength of the HCS with topping concrete in relation to interface roughness direction, the horizontal shear strength (*v_h_*) of each specimen was calculated using horizontal shear force at the interface based on experimental results as shown in Equation (8). Due to the equilibrium of forces, horizontal shear force is the same as the compressive force (*C*) or tensile force (*T*) if the interface is in the tensile area in consideration of equivalent stress block depth (a). However, if the interface is in the compressive area, it can be calculated using the compressive force (*C_c_*) acting on topping concrete. For most HCS with topping concrete, the interface is likely to be in the tensile area because the equivalent stress block depth coincides with the topping concrete layer.

(8) $$\upsilon_{h}=\frac{F_{h}}{b_{v}l_{vh}}$$

here, $F_{h}$ is the horizontal shear force of interface and $l_{vh}$ is the horizontal transfer length.

Table 4 compares the horizontal shear strength during flexural failure at the interface, obtained from the results of flexural tests on HCS with topping concrete, to horizontal shear strength requirements specified in KCI 2017 [23], ACI 318-19 [24], the PCI Design Handbook [25], and EC 2 [26].

As shown in Table 4, the horizontal shear strength acting at the interface during the flexural failure, obtained from the interfacial horizontal shear force of HCS with topping concrete, was 1.04 times larger than the horizontal shear strength proposed in KCI 2017, ACI 318-19, and the PCI Design Handbook. That is, horizontal shear strength was satisfied by interface roughness alone, even without the use of shear connectors. Compared to requirements of EC 2, which provides specific criteria on interface treatment and indented construction joint shape, the H320 series had a horizontal shear strength higher by 1.12−1.21 times. That is, the requirements were satisfied by interface roughness alone, regardless of interface roughness direction or shape of the indented construction joint. The horizontal shear strengths of the H200 series and H400 series were 0.73−0.81 and 0.82−0.90 times that of the requirement. This was a shortage by 27% at most and fell below 3% when considering the strength decrease coefficient. However, all specimens avoided horizontal shear failure before flexural failure and satisfied the flexural strength requirement, thus satisfying the horizontal shear strength requirement at the interface for the composite connection.

**Figure 11:**
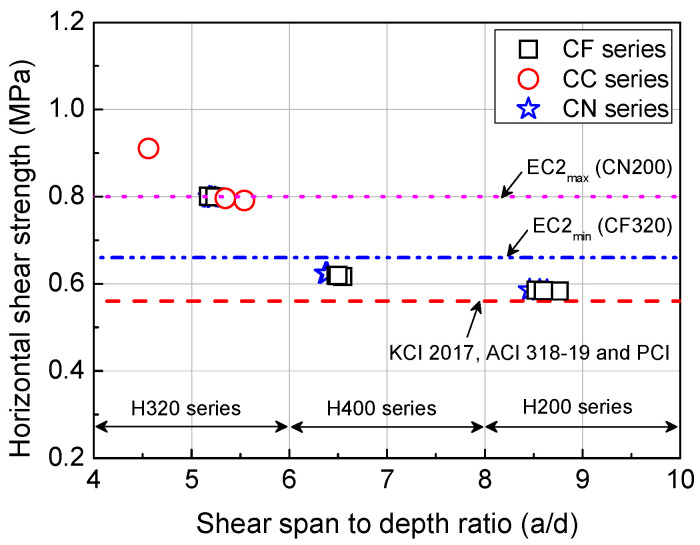
Comparison of horizontal shear strength according to shear span to depth ratio of HCS with topping concrete.

The experimental results showed that the CC series and CN series, with interface roughness achieved by applying grooves in the member length direction, had superior deformation performance, flexural strength, and structural performance compared to the CF series, in which grooves were applied in the member width direction. The application of interface roughness in the lengthwise direction is presumed to facilitate force transfer, but further research should be conducted using diverse variables for more accurate prediction. Another possible direction is to explore methods of evaluating the horizontal shear strength of specimens with interface roughness applied in the lengthwise direction. As shown in Figure 11, the horizontal shear strength acting at the interface during the flexural failure decreased with increasing shear span to depth ratio (*a/d*) regardless of interface roughness direction and shape. If the flexural behavior is dominant, it is considered necessary to secure sufficient horizontal shear strength for fully composite.

## 5. Conclusions

To evaluate the structural performance of the HCS with topping concrete, this study conducted flexural tests using the cross-sectional height and interface roughness (length and width directions) as variables. Comparing the experimental results to predictions based on design criteria, the following conclusions were obtained.

- The HCS with topping concrete exhibited ductile flexural behavior up to the peak load regardless of the cross-sectional height and direction of interface roughness. For the H200 series and H320 series, specimens with interface roughness applied in the member length direction regardless of interface roughness area had deformation higher by about 11–27% at peak load than specimens with interface roughness applied in the width (horizontal) direction. As such, the peak deflection of the HCS with topping concrete can be improved by applying interface roughness in the length direction of members and increasing the roughness area.
- Comparing the crack moment and flexural strength of the HCS with topping concrete to predictions based on KCI 2017 and ACI 318-19, the experimental-to-predicted ratios averaged 0.91−1.11 for crack moment and 0.98-1.08 for flexural strength. The predictions were fairly accurate for HCS with topping concrete, regardless of the cross-sectional height and interface roughness. Moreover, the flexural strength values obtained from experiments were higher than the crack moment values by 1.64−1.93 on average, thereby satisfying the requirement of flexural moment having to be at least 1.2 times larger than crack moment in order to induce ductile failure, as specified in KCI 2017 and ACI 318-19.
- The flexural strength of HCS with topping concrete was about 1–7% higher on average in the CN series, which applied grooves in the length direction of the members, compared to the CF series in which interface roughness was applied in the width direction of the members. When interface roughness is applied in the length direction, the structural performance requirement can be met even if the number of grooves is reduced by about 28%.
- The horizontal shear strength acting at the interface during the flexural failure, obtained from the interfacial horizontal shear force of HCS with topping concrete, was 1.04 times larger than the horizontal shear strength proposed in the design codes (except EC 2), such as KCI 2017, ACI 318-19, and the PCI Design Handbook. All specimens avoided horizontal shear failure before flexural failure and satisfied the flexural strength requirement. The required horizontal shear strength for composite connections specified in EC 2 is also presumed to have been satisfied, thus satisfying the horizontal shear strength requirement at the interface for composite connections. Further research should be conducted using diverse variables to achieve outstanding structural performance even with interface roughness applied in the lengthwise direction. A more rational method of evaluating horizontal shear strength should be developed, as well.

## Figures and Tables

**Table 1:** Interfacial horizontal shear strength specified in EC 2 [8,26].

Surface Type		Compressive Strength of Topping Concrete (MPa)	
		25	40
Very smooth	A surface cast against steel, plastic, or specially prepared wooden molds	0.30	0.41
Smooth	A slipformed or extruded surface, or a free surface left without further treatment after vibration	0.42	0.57
Rough	A surface with at least 3 mm roughness at about 40 mm spacing, achieved by raking, exposing of aggregate, or other methods giving an equivalent behavior	0.54	0.74
Indented	A surface with indentations complying with Figure 1	0.60	0.82

**Table 2:** Properties of specimens.

Specimens	*f’c* (MPa)		Size (mm)			Prestressing Strand		*a/d*	H (mm)	N
	HCS	Topping	*h*	*b*	*l*	Bottom	Top			
CF200	56.6	38.9	200	1200	5000	7-Φ12.7	2-Φ9.5	8.47	80	3
CN200										3
CF320	62.6	33.7	320			10-Φ12.7	2-Φ9.5	5.19 4.65 *	120	3
CC320	61.4									3
CN320	51.8	32.1								3
CF400	51.7	35.9	400		7000	10-Φ12.7	3-Φ12.7	6.06		3
CN400										3

* Shear span to depth ratio of CC320-1 specimen. *f’c*: compressive strength of concrete, *h*: height of section, *b*: width of section, *l*: length, *a/d*: shear span to depth ratio, H: height of topping concrete, N: number of specimens.

**Table 3:** Comparison between analytical and experimental results.

Specimens		*f’c* (MPa)	H (mm)	Experimental Results				Analytical Results (KCI 2017&ACI 318-19)			Exp. /Ana.
				*P_cr_* (kN)	*δ_cr_* (mm)	*P_peak_* (kN)	*δ_peak_* (mm)	*M_cr_* (kN∙m)	*M_n_* (kN∙m)	*M_cr, exp_* */M_cr, ana_*	*M_peak, exp_* */M_n, ana_*
CF200	1	*f’_c,HCS_* = 56.6 *f’_c,T_* = 38.9	71.8	148.7	5.39	264.7	61.08	161.1	267.7	0.96	1.03
	2		78.3	155.7	5.80	284.7	100.75	166.3	275.9	0.97	1.07
	3		76.3	154.2	5.36	287.2	83.87	164.7	273.4	0.97	1.09
Mean				152.9	5.52	278.9	81.90	164.0	272.3	0.97	1.06
CN200	1	*f’_c,HCS_* = 56.6 *f’_c,T_* = 38.9	77.3	147.2	5.67	283.2	108.58	165.5	274.7	0.92	1.07
	2		75.2	140.7	5.60	289.2	115.77	163.8	272.0	0.89	1.10
	3		80.3	153.2	5.83	279.2	86.77	167.9	278.5	0.95	1.04
Mean				147.0	5.70	283.9	103.71	165.7	275.1	0.92	1.07
CF320	1	*f’_c,HCS_* = 62.6 *f’_c,T_* = 33.7	119.5	335.0	5.30	585.5	24.02	407.3	634.0	0.85	0.96
	3		121.0	348.0	5.16	579.5	22.99	409.3	636.8	0.88	0.94
	2		116.0	381.5	5.01	622.5	26.68	402.7	627.7	0.98	1.03
Mean				354.8	5.16	595.8	24.56	406.4	632.8	0.91	0.98
CC320	1	*f’_c,HCS_* = 61.4 *f’_c,T_* = 33.7	120.0	441.0	5.32	668.0	27.22	407.1	635.0	0.99	0.96
	2		94.3	353.0	5.19	585.5	28.02	373.4	588.1	0.98	1.03
	3		108.0	353.5	5.20	640.0	36.41	391.2	613.1	0.94	1.08
Mean				382.5	5.24	631.2	30.55	390.6	612.1	0.97	1.03
CN320	1	*f’_c,HCS_* = 51.8 *f’_c,T_* = 32.1	120.3	361.0	4.29	633.5	27.51	399.3	632.1	0.94	1.04
	2		116.0	358.0	4.01	607.0	23.15	393.7	624.2	0.94	1.01
	3		118.8	370.5	4.08	660.0	30.94	397.4	629.3	0.97	1.09
Mean				363.2	4.13	633.5	27.20	396.8	628.5	0.95	1.05
CF400	1	*f’_c,HCS_* = 51.7 *f’_c,T_* = 35.9	93.2	301.7	7.27	501.3	50.74	433.1	727.2	1.04	1.03
	2		98.0	298.8	5.86	532.0	60.34	438.6	735.9	1.02	1.08
	3		96.5	285.9	5.70	520.8	70.40	436.9	733.2	0.98	1.07
Mean				295.5	6.28	518.0	60.49	436.2	732.1	1.02	1.06
CN400	1	*f’_c,HCS_* = 51.7 *f’_c,T_* = 35.9	105.8	332.4	6.12	537.4	58.44	447.5	750.2	1.11	1.07
	2 *		112.0	-	-	-	-	-	-	-	-
	3		104.5	326.2	6.27	542.4	57.82	446.0	747.8	1.10	1.09
Mean				329.3	6.20	539.9	58.13	446.8	749.0	1.11	1.08

* The CN400-2 specimen is excluded from comparison due to horizontal shear cracking that occurred before the experiment. *P_cr_*: load of at cracking, *δ_cr_*: deflection of at cracking, *P_peak_*: load of at peak; *δ_peak_*: deflection of at peak, *M_cr_*: moment of at cracking, *M_n_*: nominal moment by analytical results, *M_peak_*: moment of at peak.

**Table 4:** Comparison of horizontal shear strength.

Specimens		*f’_c_* (MPa)	Joint Surface		Experimental Results		Analytical Results		Exp./Ana.	
			*b_v_* (mm)	*l_vh_* (mm)	*F_h_* (kN)	*υ_h, exp_* (MPa)	*υ_nh, KCI_* (*υ_nh, ACI&PCI_*) (MPa)	*υ_nh, EC 2_* (MPa)	*υ_h, exp_/υ_nh, KCI_* (*υ_nh, ACI&PCI_*)	*υ_h, exp_* */υ_nh, EC 2_*
CF200	1	*f’_c,HCS_* = 56.6 *f’_c,T_* = 38.9	1200	2075	1452	0.583	0.56 (0.55)	0.72	1.04(1.06)	0.81
	2				1456	0.585			1.04(1.06)	0.81
	3				1456	0.584			1.04(1.06)	0.81
Mean					1455	0.584	-	-	1.04(1.06)	0.81
CN200	1	*f’_c,HCS_* = 56.6 *f’_c,T_* = 38.9	1200	2075	1456	0.585	0.56 (0.55)	0.80	1.04(1.06)	0.73
	2				1455	0.584			1.04(1.06)	0.73
	3				1457	0.585			1.05(1.06)	0.73
Mean					1456	0.585	-	-	1.04(1.06)	0.73
CF320	1	*f’_c,HCS_* = 62.6 *f’_c,T_* = 33.7	1200	2075	1993	0.800	0.56 (0.55)	0.66	1.43(1.45)	1.21
	3				1994	0.801			1.43(1.46)	1.21
	2				1990	0.799			1.43(1.45)	1.21
Mean					1992	0.800	-	-	1.43(1.45)	1.21
CC320	1	*f’_c,HCS_* = 61.4 *f’_c,T_* = 33.7	1200	1825	1993	0.910	0.56 (0.55)	0.73	1.63(1.65)	1.25
	2			2075	1969	0.791			1.41(1.44)	1.08
	3				1982	0.796			1.42(1.45)	1.09
Mean					1982	0.832	-	-	1.49(1.51)	1.14
CN320	1	*f’_c,HCS_* = 51.8 *f’_c,T_* = 32.1	1200	2075	1988	0.798	0.56 (0.55)	0.71	1.43(1.45)	1.12
	2				1984	0.797			1.42(1.45)	1.12
	3				1987	0.798			1.42(1.45)	1.12
Mean					1986	0.798	-	-	1.42(1.45)	1.12
CF400	1	*f’_c,HCS_* = 51.7 *f’_c,T_* = 35.9	1200	3000	2219	0.616	0.56 (0.55)	0.69	1.10(1.12)	0.89
	2				2229	0.619			1.11(1.13)	0.90
	3				2226	0.618			1.10(1.12)	0.90
Mean					2225	0.618	-	-	1.10(1.12)	0.90
CN400	1	*f’_c,HCS_* = 51.7 *f’_c,T_* = 35.9	1200	3000	2245	0.624	0.56 (0.55)	0.76	1.11(1.13)	0.82
	2 *				-	-			-	-
	3				2242	0.623			1.11(1.13)	0.82
Mean					2243	0.623	-	-	1.11(1.13)	0.82

* The CN400-2 specimen is excluded from comparison due to horizontal shear cracking that occurred before the experiment.

## Data Availability

The data presented in this study are available on request from the corresponding author.
